# Physical activity initiated by employer induces improvements in a novel set of biomarkers of inflammation: an 8-week follow-up study

**DOI:** 10.1007/s00421-016-3533-5

**Published:** 2017-02-09

**Authors:** Lars-Kristian Lunde, Øivind Skare, Hans C. D. Aass, Asgeir Mamen, Elín Einarsdóttir, Bente Ulvestad, Marit Skogstad

**Affiliations:** 1 Department of Occupational Medicine and Epidemiology, National Institute of Occupational Health, Box 8149 Dep., 0033 Oslo, Norway; 2 Department of Work Psychology and Physiology, National Institute of Occupational Health, Box 8149 Dep., 0033 Oslo, Norway; 3 The Blood Cell Research Group, Section of Research, Department of Medical Biochemistry, Oslo University Hospital, Ullevål, Norway; 4 Norwegian School of Health Sciences, Kristiania University College, Box 1190 Sentrum, 0107 Oslo, Norway; 5 Department of Chemical and Biological Work Environment, National Institute of Occupational Health, Box 8149 Dep., 0033 Oslo, Norway

**Keywords:** Biomarkers of inflammation, Cardiovascular disease, Cytokines, Disease prevention, Health promotion, Physical activity

## Abstract

**Purpose:**

We investigated the level of pro- and anti-inflammatory biomarkers before and after 8 weeks of unsupervised physical activity (PA) initiated by employer.

**Methods:**

During autumn 2014, background data, blood samples and self-reported exercise level were collected from 76 men and 41 women in a Norwegian road maintenance company. Monocyte chemoattractant protein-1 (MCP-1), tumor necrosis factor-alpha (TNF-α), interleukin-6 (IL-6), leptin, adiponectin, p-selectin and CD40 ligand (CD40L) were analyzed. ${\dot V}{{\rm{O}}_{{\rm{2max}}}}$ was measured in a subgroup of 50 subjects.

**Results:**

With reference point of exercise ≤1 time/week, we found that participants who exercised 2–3 times/week had higher ${\dot V}{{\rm{O}}_{{\rm{2max}}}}$ values (5.6 mL kg^−1^ min^−1^; 95% CI [1.3, 9.9]). MCP-1 was lower in those who exercised ≥ 4 times/week (−81.98 pg/ml [−142.9, −21.0]). IL-6 and p-selectin levels were lower in females who exercised ≥4 times/week (−1.04 pg/ml [−2.04, −0.03] and −13.75 ng/ml [−24.03, −3.48]). Leptin was lower in participants who exercised 2–3 times/week (−0.39 µg/ml ln [−0.68, −0.09]) and ≥4 times/week (−0.69 µg/ml ln [−1.10, −0.28]). During follow-up, ${\dot V}{{\rm{O}}_{{\rm{2max}}}}$ increased (2.9 mL kg^−1^ min^−1^ [1.5, 4.3]), while p-selectin and CD40L decreased (−2.33 ng/ml [−3.78, −0.87] and 718.14 ng/ml [−1368, −68]). MCP-1 levels decreased among men (−32.70 pg/ml [−51.21, −14.19]). A joint analysis of all biomarkers (inversed adiponectin) showed that those who exercised ≥4 times/week at baseline had lower total levels of biomarkers and that total biomarker levels decreased during follow-up.

**Conclusions:**

Exercising several times a week was associated with less inflammation compared to exercising once a week or less. During the 8-week follow-up, total levels of biomarkers of inflammation improved.

**Electronic supplementary material:**

The online version of this article (doi:10.1007/s00421-016-3533-5) contains supplementary material, which is available to authorized users.

## Introduction

Systemic inflammation may contribute to cardiovascular diseases (CVD), including coronary heart disease and stroke (Libby 2002). The development and progression of atherosclerotic CVD is a complex process, involving several inflammatory agents acting at different stages (Fleg et al. 2012). The inflammatory biomarker C-reactive protein (CRP) has, for a long time, been associated to inflammation and risk of CVD (Ridker et al. 1997). Abnormal levels of other pro- and anti-inflammatory agents such as, monocyte chemoattractant protein-1 (MCP-1), tumor necrosis factor-alpha (TNF-α), interleukin-6 (Il-6), leptin, adiponectin, CD40 ligand (CD40L) and p-selectin may also affect CVD risk (Stoner et al. 2013; Tuttolomondo et al. 2012). Inflammation is additionally linked to cancer development, and increased levels of TNF-α, IL-6, selectins, and leptin and decreased levels of adiponectin are associated with enhanced metastasis and increased risk of several cancers (Libby et al. 2011; Yamauchi et al. 2014; Winzer et al. 2011; Coupland and Parish 2014).

Studies carried out in patients with metabolic syndrome have shown an association between physical activity (PA) and CVD and general mortality (Katzmarzyk et al. 2004). High level of PA is also shown to reduce TNF-α and IL-6 in people with CVD risk (Palmefors et al. 2014) and IL-6 in patients with diabetes-2 (Hayashino et al. 2014). Exercise is shown to increase the expression of adiponectin receptors in obese individuals (Christiansen et al. 2010) and long-term endurance training may increase adiponectin levels in both obese and diabetes-2 patients (Lee and Kwak 2014; Brunelli et al. 2015). Leptin seems not to be affected by short-term bouts of exercise, but exercise for longer periods that lead to reduction in adipose tissue can lower the leptin levels (Golbidi and Laher 2014).

A 10-year follow-up of healthy individuals showed that those who were physically active at baseline had significantly lower IL-6 levels compared to those rarely active. In addition, an increase in PA by at least 2.5 h/week during the follow-up reduced these biomarkers significantly (Hamer et al. 2012).

Although MCP-1, p-selectin, and CD40L are thought to play major roles in atherosclerosis (Tuttolomondo et al. 2012), the possible effect of PA on these agents is unknown due to contradictory and/or scarce scientific evidence (Palmefors et al. 2014; Chen et al. 2014).

Employers are encouraged to promote employees’ health through PA (The European Network for Workplace Health Promotion Web site 2015). Both internet-based motivation programs (Andersen et al. 2013) and structured exercise sessions (Korshoj et al. 2015) have been shown to successfully increase the level of PA in workers. Such increase in PA may have positive effects on risk biomarkers for CVD, but there is scarce knowledge of how PA initiated at the workplace will affect such variables in generally healthy employees. CVD are considered one of the largest challenges globally, with high blood pressure causing 10.4 million deaths and 208.1 million disability-adjusted life-years in 2013 (Forouzanfar et al. 2015); thus, effective preventive strategies are important. We recently investigated associations between PA and levels of CRP, glycosylated hemoglobin, low-density lipoprotein, high-density lipoprotein, total cholesterol, blood pressure and resting heart rate under the same conditions as in the present study (Skogstad et al. 2016). Results showed that high level of PA was associated with more favorable lipid status and lower blood pressure, and that 8 weeks of increased PA initiated by employer reduced the total cholesterol and LDL.

In the present study, we hypothesized that greater levels of PA would be associated with more beneficial levels of MCP-1, TNF-α, IL-6, leptin, adiponectin, p-selectin and CD40L and that an 8-week employer initiative to increase employees PA would lead to an overall improvement in these agents. These putative predictors of health will, in this paper, be addressed by the generic term biomarkers.

## Methods

The initial study population and exercise regimen presented in this study are equal to that presented previously by Skogstad and colleagues (Skogstad et al. 2016).

### Study population

In a Norwegian road maintenance enterprise with 1498 employees, information of the PA motivation initiative by the employer was distributed via information meetings, the enterprise newspaper and intranet for two months prior to startup. Three hundred employees wished to participate and were additionally given the opportunity to participate in our study. Out of those 300, a total of 121 gave their informed written consent to participate and were included. Two of the 121 were excluded from the analyses due to medical reasons (heart surgery and prescribed antibiotics for sinusitis). For two participants, biomarker data could not be obtained, giving a total sample of 117.

### Study design

The enterprise management organized an 8-week initiative (“Dytt®”) as motivation to increase PA, running from September 15 to November 16, 2014. The participants competed against each other individually and in self-selected teams (2–8 members) in a virtual internet mountain track. Participants moved forward by registering data logged with a wristband pedometer measuring their daily steps. Step measurement was for participant motivation and PA activities were home-based, self-selected (amount of aerobic exercise and resistance training was by own choice), and performed unsupervised. Participants had access to their own and their team’s progress, in addition to best daily performers and overall leaders. Activities that were poorly measured by the pedometer (resistance training, spinning) could be converted into steps and registered. The best individual performer and team received a prize at the end of the period. The 117 participants in the present study met for medical examinations before and after the eight weeks, which included questionnaires, blood samples and standardized blood pressure measurements and resting heart rate measurements. A subgroup of 50 participants were tested for ${\dot V}{{\rm{O}}_{{\rm{2max}}}}$ in connection with their medical examination.

### Questionnaire

Subjects were asked for medical history including treatment at hospital for cancer, diabetes, cardiovascular disease or chronic obstructive lung disease and current use of prescribed medicines. Age, height and weight were registered and subjects were asked for education level, smoking status and number of days with PA per week. We used the question “How often do you normally exercise?” to assess the level of PA, with the response alternatives: never, less than once per week, once per week, two to three times per week or four or more times per week (Holth et al. 2008). To obtain groups containing a reasonable amount of participants, the answers were categorized into once a week or less, 2–3 times per week and 4 or more times per week. In the follow-up questionnaire, the participants were additionally asked for the daily average number of steps recorded on their online profile during the PA period, any change in the duration of low-intensity (walking, golf, garden work) and/or high-intensity (jogging, spinning, playing tennis) activity. At follow-up, subjects were additionally asked if they had made any changes in their diet, intake of alcohol or smoking habits during the course of the program.

### Aerobic fitness

A standardized graded test on a cycle ergometer (Monark 874 E, Monark Exercise AB, Vansbro, Sweden) in combination with a Cosmed K4b2 metabolism analyzer (Cosmed Srl, Rome, Italy) determined physical fitness in terms of ${\dot V}{{\rm{O}}_{{\rm{2max}}}}$. Participants started with a load of 70 W and a cadence of 70 revolutions per minute (RPM). Load was increased by 35 W every minute until the subjects’ cadence fell below 65 RPM and were considered exhausted. The 30-s averaged interval with the highest measurements was used to calculate ${\dot V}{{\rm{O}}_{{\rm{2max}}}}$. This parameter was used as an indication of exercise impact on fitness, not as a main outcome.

### Blood collection and serum preparation

Blood was sampled from non-fasting subjects at the same time of day before and after the PA period. Blood sampling was scheduled to be performed before noon if possible. Care was taken to ensure that each subject was tested at the same time at baseline and follow-up. We encouraged subjects not to participate in any strenuous physical activity 24 h prior to sampling. The samples were left for at least 30 min and maximum 60 min at room temperature prior to 1000×*g* centrifugation for 15 min, and being aliquoted into 500-μl volumes in 1.8-mL Nunc tubes and stored at −80 °C.

### Serum analysis

Serum levels of MCP-1, IL-6, TNF-α, leptin, p-selectin and CD40L were analyzed with a Luminex IS 100 (Bio Rad, Hercules, CA, USA) powered with the Bio-Plex Manager software version 6.0 build 617. All samples were blinded (given a four-digit code) and distributed randomly assayed on 12 different plates. Samples were analyzed in duplicate and the average intra-assay %CV for all samples was determined; MCP-1 (5.7), IL-6 (4.0), p-selectin (2.0), CD40L (3.4), leptin (3.4), TNF-α (3.7) and adiponectin (2.7). An in-house longitudinal control was used on each plate (*n* = 7) to determine the inter-assay %CV; MCP-1 (10.0), IL-6 (6.8), p-selectin (8.3), CD40L (11.8), leptin (14.5) and TNF-α (7.5). See supplementary appendix A for the full description of the standardized method used.

### Statistical analysis

Unadjusted linear regression was used to investigate the baseline differences between dropouts and non-dropouts.

Each biomarker and ${\dot V}{{\rm{O}}_{{\rm{2max}}}}$ was analyzed with a linear mixed model. Using baseline exercise and time (baseline and follow-up) as explanatory variables, we estimated both the association between baseline exercise and the biomarker/${\dot V}{{\rm{O}}_{{\rm{2max}}}}$, and the change (from baseline to follow-up) in biomarkers during follow-up. We used the lowest category of exercise as the reference value. The leptin values were ln-transformed prior to analysis to give a better fit to the normal distribution.

We also investigated the effect of exercise (association and change) on all biomarkers by implementing a joint biomarker model. Prior to analysis, each biomarker was normalized to a mean = 0 and SD = 1. Since increased adiponectin levels are considered favorable, this biomarker was reversed by multiplying its values with −1. A reduction for the normalized joint biomarker value would then be favorable. A linear mixed model was applied to this normalized joint biomarker as outcome variable.

Two observations for IL-6 with standardized residuals exceeding 3.0 were considered outliers and removed. All mixed-model analyses were adjusted for age, sex, smoking status and education, and stratified by sex. All available observations were included in the analyses, including those from participants with incomplete observations. For the joint model, based on likelihood-ratio tests, we chose a model with common effect of exercise, time, education and smoking, while the intercepts and the sex and age effects were allowed to vary with biomarker.

In the analyses of ${\dot V}{{\rm{O}}_{{\rm{2max}}}}$, a random intercept for subject was added. We took plate variation into account in addition to the between-subject variation by including crossed random intercepts for plate and subject. For the joint biomarker model, a random intercept was added for biomarker, nested in subject, and also, a random slope was included for subject. See supplementary appendix B for a detailed description of the R and Stata implementation of the joint and separate biomarker analyses.

BMI may work as a mediator for the effect of PA. To assess the impact of BMI on biomarkers, we reran the mixed-model analyses with an additional adjustment for BMI. Likewise, we used interaction analysis to investigate if the change from baseline to follow-up differed with the above-mentioned covariates. Statistical analyses were done using R (version 3.2.2) and Stata (version 14.1).

## Results

### Participant characteristics and loss to follow-up

Out of the 117 participating, 30 were road workers (26%), while 87 were office workers (74%). At baseline, five individuals were classified as having metabolic syndrome based on commonly agreed upon criteria (Alberti et al. 2009). See Table 1 for baseline characteristics.

**Table 1:** Characteristics at baseline stratified on gender and exercise category

Variable	Female			Male		
	≤1 time/week (*N* = 16)	2–3 times/week (*N* = 20)	≥4 times/week (*N* = 5)	≤1 time/week (*N* = 28)	2–3 times/week (*N* = 35)	≥4 times/week (*N* = 13)
	Mean (SD)	Mean (SD)	Mean (SD)	Mean (SD)	Mean (SD)	Mean (SD)
Age (years)	42.6 (12.9)	43.0 (12.1)	47.0 (12.9)	40.3 (10)	43.3 (12.1)	41.7 (15.2)
Height (cm)	167 (7.3)	168.5 (5.2)	168.7 (2.5)	180 (7.1)	181.1 (7)	181.1 (5.3)
BMI (kg/m^2^)^a^	24.9 (3.7)	23.9 (2.7)	23.6 (2.5)	27.2 (5.4)	26 (3)	25.2 (2.7)
SBP (mmHg)	114.6 (16)	115.5 (16.6)	120.9 (15.1)	123.6 (10.8)	125.1 (12.6)	118.2 (14)
DBP (mmHg)	74.6 (8.1)	73.3 (7.4)	74.7 (9.2)	79.3 (6.7)	80.8 (8.2)	72.9 (11)
Resting HR (bpm)	68.3 (6.7)	64.3 (9.8)	65 (8.3)	66.4 (9.5)	62 (9.7)	61.4 (8)
${\dot V}{{\rm{O}}_{{\rm{2max}}}}$ ^b^	30.5 (4.3)	37.5 (8.4)	26.5 (12.3)	35.2 (5.1)	41 (6.3)	42.5 (9.6)
MCP-1 (pg/mL)	304.7 (84.3)	288.8 (90.3)	258.2 (83.7)	359.3 (141)	352.1 (135.1)	275.1 (83.3)
TNF-α (pg/mL)	4.3 (3.6)	2.8 (3.8)	1.7 (2.4)	7.7 (4)	6.5 (4.5)	7.8 (5.4)
IL-6 (pg/mL)	2.6 (1.3)	2.3 (1.5)	1.8 (0.5)	2.6 (1.3)	3.1 (2)	3.1 (2.5)
Leptin (μg/mL)	23.2 (21)	12 (7.8)	14.3 (11.4)	8.2 (6.8)	6.5 (5.8)	4 (3.4)
Adiponectin (μg/mL)	10.7 (4.1)	11.3 (3.8)	11.2 (3.3)	7.7 (2.8)	7.6 (2.9)	8.5 (4)
P-selectin (ng/mL)	43.2 (8.2)	42.1 (12.2)	29.5 (11)	48 (17.4)	51 (17.5)	47.1 (17.6)
CD40L (pg/mL)	5569.6 (2285.6)	4642.3 (2207.8)	3499.2 (1605.1)	4711.7 (2285.1)	4944.8 (3166.7)	3488.8 (3040.1)
Smokers (%)^c^	0 (0%)	0 (0%)	0 (0%)	7 (25%)	4 (11%)	1 (8%)
Uni/College (%)^c^	12 (75%)	18 (90%)	5 (100%)	13 (46%)	20 (57%)	7 (54%)
White collar (%)^c^	16 (100%)	20 (100%)	5 (100%)	15 (54%)	26 (74%)	6 (46%)

*BMI* body mass index, *SBP* systolic blood pressure, *DBP* diastolic blood pressure, *HR* heart rate

^a^ *N* = 19 for females exercising 2–3 times/week

^b^ *N* = 6, 10, 3 (females) and 11, 14, 6 (males) for the three exercise categories, respectively

^c^Number of observations and percentage

Ninety-eight out of the 117 examined at baseline were available at follow-up, and 19 were not (quit job, refused health examination). Dropouts did not differ from compliers in the reported amount of weekly exercise at baseline or any other variable. Two participants tested for ${\dot V}{{\rm{O}}_{{\rm{2max}}}}$ did not provide blood samples. Thirteen of the 50 tested for ${\dot V}{{\rm{O}}_{{\rm{2max}}}}$ at baseline were not tested at follow-up (quit job, refused health examination). Compared to the compliers, these 13 had lower adiponectin values (7.7 vs. 10.9 μg/mL, *P* = 0.015). One person was only tested for ${\dot V}{{\rm{O}}_{{\rm{2max}}}}$ at follow-up. Four of the five classified as having metabolic syndrome at baseline were lost to follow-up.

### Exercise levels and fitness

At baseline, 44 persons exercised once a week or less, while 55 reported 2–3 times a week. Eighteen stated that they exercised four times or more. Fifty-two per cent increased the exercise frequency during the PA period, and at follow-up, 12 exercised ≤1 time/week, 58 2–3 times/week and 28 exercised ≥4 times/week. On average, male participants increased the daily low-intensity activity (walking) by 13.7 min (SD 29.4) and high-intensity activity (running) by 8.3 min (SD 18.2). For females, the corresponding increases were 14.5 min (SD 17.3) and 9.8 min (SD 13.4). Ninety-one of the 98 observed at follow-up uploaded their steps to the Dytt® page online and had a median of 16,228 steps per day.

With exercise ≤1 time/week as reference, participants that exercised 2–3 times/week had significantly higher ${\dot V}{{\rm{O}}_{{\rm{2max}}}}$ values (5.6 mL kg^−1^ min^−1^; *P* = 0.0149). The ${\dot V}{{\rm{O}}_{{\rm{2max}}}}$ values for those that exercised ≥4 times/week were of almost similar magnitude but had a lower *n* and were not significant (4.7 mL kg^−1^ min^−1^; *P* = 0.10). ${\dot V}{{\rm{O}}_{{\rm{2max}}}}$ increased by 2.9 mL kg^−1^ min^−1^ (*P* = 0.0003) from baseline to follow-up.

### Association between exercise levels and biomarkers

*MCP-1* levels in all subjects were significantly lower in those that exercised ≥4 times/week (−81.98 pg/ml; *P* = 0.0086), compared to those that exercised ≤1 time per week. *IL-6* levels were significantly lower in females with an exercise level ≥4 times/week (−1.04 pg/ml; *P* = 0.044). *Leptin* levels were significantly lower in participants that exercised 2–3 times per week (−0.39 µg/ml ln; *P* = 0.011) and ≥4 times/week (−0.69 µg/ml ln; *P* = 0.0012). *P-selectin* was significantly lower in women who exercised ≥4 times/week (−13.75 ng/ml; *P* = 0.0094). See Tables 2, 3 and 4.

**Table 2:** The effect of exercise (times per week) and time (change from baseline to follow-up) on MCP-1 and TNF-α

Outcome	Covariate	Category	Females (*N* = 76/41)				Males (*N* = 136/76)				All (212/117)			
			*β*	95% CI		*P*	β	95% CI		*P*	*β*	95% CI		*P*
MCP-1	Exercise	2–3 t/week	−13.30	−71.47	44.87	0.65	−9.02	−71.03	53.00	0.77	−17.15	−60.94	26.64	0.44
(pg/mL)		≥ 4 t/week	−37.34	−128.44	53.75	0.42	−90.81	−172.28	−9.35	0.029	−81.98	−142.92	−21.04	0.0086
	Time	Follow-up	19.18	−7.05	45.42	0.15	−32.70	−51.21	−14.19	0.00065	−13.44	−29.38	2.50	0.098
	Age		18.66	−5.37	42.69	0.13	18.16	−5.92	42.25	0.14	20.45	3.37	37.52	0.019
	Gender	Female	∙	∙	∙	∙	∙	∙	∙	∙	−41.06	−86.26	4.14	0.075
	Education	College/Uni	−3.90	−83.57	75.78	0.92	19.49	−35.67	74.64	0.49	15.04	−29.09	59.17	0.5
	Smoking		∙	∙	∙	∙	−6.88	−84.28	70.53	0.86	−9.47	−77.51	58.58	0.78
TNF-α	Exercise	2–3 t/week	−1.39	−3.84	1.05	0.26	−0.72	−2.78	1.35	0.49	−0.72	−2.29	0.86	0.37
(pg/mL)		≥ 4 t/week	−1.57	−5.38	2.24	0.41	1.05	−1.68	3.79	0.45	0.36	−1.83	2.56	0.74
	Time	Follow-up	0.46	−0.50	1.43	0.34	−0.69	−1.42	0.03	0.062	−0.26	−0.85	0.32	0.37
	Age		0.53	−0.45	1.51	0.29	0.40	−0.42	1.22	0.33	0.36	−0.25	0.98	0.25
	Gender	Female	∙	∙	∙	∙	∙	∙	∙	∙	−2.77	−4.40	−1.15	0.00091
	Education	College/Uni	0.76	−2.67	4.20	0.66	−2.00	−3.84	−0.17	0.033	−1.53	−3.11	0.06	0.059
	Smoking		∙	∙	∙	∙	2.39	−0.19	4.97	0.069	2.34	−0.10	4.79	0.06

Model is adjusted for age, gender, education (high school/college or university), and smoking (no/yes). Intercept value not shown. *N* indicates number of observations and subjects. Reference category for exercise is exercise ≤1 time/week. The variable time represents change from baseline to follow-up, where the reference is baseline

**Table 3:** The effect of exercise (times per week) and time (change from baseline to follow-up) on IL-6, leptin and adiponectin

Outcome	Covariate	Category	Females (*N* = 76/41)				Males (*N* = 136/76)				All (212/117)			
			*β*	95% CI		*P*	*β*	95% CI		*P*	*β*	95% CI		*P*
IL-6	Exercise	2–3 t/week	−0.22	−0.86	0.42	0.49	0.07	−0.55	0.69	0.82	−0.04	−0.49	0.41	0.85
(pg/mL)		≥ 4 t/week	−1.04	−2.04	−0.03	0.044	0.24	−0.58	1.06	0.57	−0.06	−0.69	0.57	0.85
	Time	Follow-up	−0.24	−0.57	0.09	0.15	−0.07	−0.34	0.20	0.62	−0.13	−0.33	0.08	0.24
	Age		0.10	−0.18	0.37	0.49	0.29	0.04	0.54	0.022	0.22	0.05	0.40	0.014
	Gender	Female	∙	∙	∙	∙	∙	∙	∙	∙	−0.31	−0.77	0.16	0.19
	Education	College/Uni	−0.15	−1.01	0.71	0.73	−0.65	−1.20	−0.11	0.02	−0.50	−0.95	−0.05	0.03
	Smoking		∙	∙	∙	∙	0.80	0.03	1.56	0.042	0.83	0.13	1.53	0.02
Leptin ln	Exercise	2–3 t/week	−0.46	−0.95	0.02	0.061	−0.35	−0.74	0.04	0.081	−0.39	−0.68	−0.09	0.011
(μg/mL)		≥ 4 t/week	−0.36	−1.12	0.40	0.35	−0.79	−1.31	−0.28	0.0027	−0.69	−1.10	−0.28	0.0012
	Time	Follow-up	0.02	−0.08	0.13	0.63	0.00	−0.09	0.09	0.95	0.01	−0.06	0.08	0.82
	Age		0.10	−0.09	0.30	0.29	0.14	−0.01	0.29	0.075	0.13	0.01	0.25	0.027
	Gender	Female	∙	∙	∙	∙	∙	∙	∙	∙	1.01	0.70	1.31	<0.0001
	Education	College/Uni	−0.32	−1.00	0.37	0.36	−0.37	−0.72	−0.02	0.039	−0.33	−0.63	−0.03	0.03
	Smoking		∙	∙	∙	∙	−0.14	−0.63	0.35	0.57	−0.14	−0.60	0.33	0.57
Adiponectin	Exercise	2–3 t/week	0.60	−1.80	2.99	0.62	−0.54	−2.07	0.99	0.49	−0.20	−1.51	1.11	0.77
(μg/mL)		≥ 4 t/week	0.03	−3.71	3.77	0.99	0.36	−1.66	2.38	0.73	0.57	−1.25	2.39	0.54
	Time	Follow-up	0.05	−0.41	0.50	0.83	0.15	−0.14	0.45	0.31	0.12	−0.13	0.36	0.36
	Age		1.15	0.17	2.12	0.022	−0.25	−0.85	0.36	0.42	0.32	−0.19	0.83	0.22
	Gender	Female	∙	∙	∙	∙	∙	∙	∙	∙	3.17	1.82	4.52	<0.0001
	Education	College/Uni	1.00	−2.36	4.36	0.55	0.36	−1.00	1.72	0.6	0.41	−0.90	1.73	0.54
	Smoking		∙	∙	∙	∙	0.47	−1.45	2.39	0.63	0.38	−1.66	2.42	0.71

Model is adjusted for age, gender, education (high school/college or university), and smoking (no/yes). Intercept value not shown. *N* indicates number of observations and subjects. Reference category for exercise is exercise ≤1 time/week. The variable time represents change from baseline to follow-up, where the reference is baseline

**Table 4:** The effect of exercise (times per week) and time (change from baseline to follow-up) on p-selectin and CD40L

Outcome	Covariate	Category	Females (*N* = 76/41)				Males (*N* = 136/76)				All (212/117)			
			*β*	95% CI		*P*	β	95% CI		*P*	β	95% CI		*P*
P-selectin	Exercise	2–3 t/week	0.71	−5.84	7.26	0.83	1.39	−6.27	9.05	0.72	1.42	−3.96	6.80	0.6
(ng/mL)		≥ 4 t/week	−13.75	−24.03	−3.48	0.0094	−1.68	−11.78	8.43	0.74	−4.38	−11.89	3.12	0.25
	Time	Follow-up	−0.87	−3.43	1.69	0.5	−3.11	−4.89	−1.33	0.00075	−2.33	−3.78	−0.87	0.0019
	Age		1.97	−0.76	4.71	0.15	3.34	0.29	6.38	0.032	2.88	0.76	4.99	0.0078
	Gender	Female	∙	∙	∙	∙	∙	∙	∙	∙	−9.70	−15.24	−4.16	0.00068
	Education	College/Uni	−0.39	−9.30	8.52	0.93	−1.43	−8.21	5.36	0.68	−1.14	−6.55	4.27	0.68
	Smoking		∙	∙	∙	∙	0.27	−9.31	9.85	0.96	0.51	−7.87	8.89	0.91
CD40L	Exercise	2–3 t/week	−142.88	−1350.23	1064.48	0.81	−394.78	−1437.37	647.80	0.46	−163.47	−937.25	610.30	0.68
(pg/mL)		≥ 4 t/week	−1102.54	−3001.68	796.61	0.25	−1075.76	−2485.72	334.20	0.13	−903.74	−2000.46	192.98	0.11
	Time	Follow-up	−473.18	−1544.67	598.31	0.38	−822.20	−1633.57	−10.84	0.047	−718.14	−1368.26	−68.01	0.031
	Age		175.20	−326.21	676.61	0.49	118.09	−301.04	537.22	0.58	129.72	−173.26	432.70	0.4
	Gender	Female	∙	∙	∙	∙	∙	∙	∙	∙	72.61	−723.11	868.32	0.86
	Education	College/Uni	846.79	−763.65	2457.23	0.3	−568.36	−1503.65	366.94	0.23	−196.96	−977.13	583.21	0.62
	Smoking		∙	∙	∙	∙	−219.97	−1510.33	1070.40	0.74	−315.98	−1509.37	877.41	0.6

Model is adjusted for age, gender, education (high school/college or university), and smoking (no/yes). Intercept value not shown. *N* indicates number of observations and subjects. Reference category for exercise is exercise ≤1 time/week. The variable time represents change from baseline to follow-up, where the reference is baseline

The joint trend model analyzing associations between PA and all biomarkers, showed that those exercising ≥4 times per week had significantly lower total level of biomarkers (−0.34; *P* = 0.0085). See Table 5.

**Table 5:** Joint analysis of all biomarkers—exercise level (times per week) and time (change from baseline to follow-up)

Covariate	Category	Females (*N* = 76/41)				Males (*N* = 136/76)				All (212/117)			
		β	95% CI		*P*	β	95% CI		*P*	β	95% CI		*P*
Exercise	2–3 times/wk	−0.170	−0.391	0.051	0.14	−0.062	−0.310	0.185	0.62	−0.089	−0.267	0.089	0.33
	≥ 4 times/wk	−0.582	−0.929	−0.235	0.0026	−0.292	−0.619	0.036	0.085	−0.341	−0.590	−0.092	0.0085
Time	Follow-up	−0.015	−0.148	0.118	0.83	−0.148	−0.225	−0.071	0.00038	−0.101	−0.171	−0.032	0.0052

The effect of exercise level and time (change from baseline to follow-up) on biomarker adjusted for age, gender, education (high school/college or university) and smoking (no/yes). *N* indicates number of observations/subjects. Reference category for exercise is exercise ≤1 time/week. The variable time represents change from baseline to follow-up, where the reference is baseline

### Changes in biomarkers between baseline and follow-up

MCP-1 levels decreased during follow-up in men (−32.70 pg/ml; *P* = 0.00065) but not in women. Both p-selectin and CD40L decreased significantly by 2.33 ng/ml (*P* = 0.0019) and 718.14 ng/ml (*P* = 0.031), respectively. See Tables 2, 3 and 4.

The joint model estimating the average change from baseline to follow-up of all biomarkers, showed a significant reduction in levels for all subjects (−0.10; *P* = 0.0052) with largest contributions from men (-0.15; *P* = 0.00038). See Table 5.

### Impact of BMI

BMI affected the associations between biomarkers and exercise only to a minor degree, and had minimal impact on the change in biomarkers during follow-up. See supplementary appendix C and D.

## Discussion

We observed that self-reported exercise several times per week was associated with more favorable levels of MCP-1, IL-6 (women), leptin and p-selectin (women). Half of the participants reported an increase in their exercise level during follow-up and a decrease in MCP-1 (men), CD40L and p-selectin was found. When analyzing the total effect of exercise on all biomarkers, we found more favorable levels of biomarkers in individuals with high levels of exercise and a significant improvement from baseline to follow-up, reflecting a mutual reduction in inflammation.

Several potential mechanisms have been proposed to explain how exercise could reduce chronic inflammation: anti-inflammatory myokine production; the alteration of adipose tissue to reduce hypoxia and inflammation; lower leukocyte adhesion in endothelial cells and systematic cytokine production and immune system effects resulting in a lower number of pro-inflammatory cells with less cytokine production (You et al. 2013).

### Physical activity, inflammation and adipokines

An abundance of MCP-1 in the vessel wall, along with hyperlipidemia, accelerates atherosclerosis (Lin et al. 2014) and may facilitate plaque rupture (Cai et al. 2015). In addition, a small increase in MCP-1 is shown to prompt insulin resistance (Sell et al. 2006). We found lower levels of MCP-1 in those with frequent PA compared to those who reported less frequent PA, and a reduction in this biomarker for men during follow-up. To our knowledge, this is the first study to suggest that regular PA reduces MCP-1 in a sample of healthy adults (Palmefors et al. 2014), with similar findings previously showed only in a limited number (*N* = 13) of young healthy individuals (Czepluch et al. 2011) or rodents (Murphy et al. 2015). For the present study, MCP-1 was the biomarker showing the most consistent gender difference, both with respect to the association with baseline PA and with the change from baseline to follow-up. There are clear sex differences in immune responses (Klein and Flanagan 2016), and a similar mechanism might explain that sex has an impact on the link between physical activity and MCP-1 levels. Normally, MCP-1 is higher in men than in women (Samnegård et al. 2009), which might partly explain gender differences in pathophysiological mechanisms of cardiovascular disease (Samnegård et al. 2009).

IL-6 promotes the initiation and progression of atheromatous plaques and stimulates the endothelium to recruit leucocytes to the intima (Christodoulidis et al. 2014). IL-6 is also known to impair insulin sensitivity (Golbidi and Laher 2014) and stimulate liver production of CRP (Libby 2002). Although the mechanisms involved are debated, studies have shown that increased circulating levels of IL-6 may increase the risk of CVD and general mortality (Khan et al. 2015; Karstoft and Pedersen 2016). Our study found that a higher level of PA is associated with lower levels of IL-6 in females. Other studies have found that the acute effect of PA is release of IL-6 into the circulation, while regular PA has shown to provide a decrease in IL-6 levels (Chen et al. 2014; Karstoft and Pedersen 2016).

Leptin can induce oxidative stress mediating a pro-inflammatory state and is a marker for obesity (You et al. 2013). We found significantly lower leptin levels in highly active people compared to less active people. Studies have previously shown that leptin levels are lower in physically fit and that long bouts of PA with increased energy expenditure, can decrease leptin (Golbidi and Laher 2014; You et al. 2013). Although it is seen as beneficial to reduce leptin levels, the clinical significance of reducing leptin is not yet clear (Hayashino et al. 2014; Korda et al. 2008).

TNF-α promotes atherosclerosis by endothelial dysfunction, promotes oxidative stress and is present in early atherosclerosis (Korda et al. 2008), in addition to being involved in dyslipidemia and insulin resistance (Christodoulidis et al. 2014). It is suggested that TNF-α is reduced by PA through the IL-6 pathways and adrenergic mechanisms (Golbidi and Laher 2014). Although regular PA may reduce TNF-α levels (Chen et al. 2014), we cannot report such findings.

We did not find any significant association between adiponectin and exercise level or change in adiponectin levels during follow-up. Adiponectin improves insulin sensitivity, glucose tolerance and lipid profile and decreases inflammation and atherosclerosis. A beneficial increase of circulating adiponectin due to PA among overweight and patients with the metabolic syndrome has been shown (Wang et la. 2015, Tjonna et al. 2008). However, in studies of subjects with normal weight, there have been inconsistent results regarding a possible association (Lee and Kwak 2014).

Adjusting for BMI did not impact the results on either individual biomarkers or on the joint effect. Thus, as found in other studies, changes in biomarkers did not seem to largely depend on changes in weight (Brunelli et al. 2015).

### Physical activity, platelet and endothelial activation

P-selectin is a transmembrane protein involved in the coagulation process, resulting in endothelial injury, thus promoting atherogenesis (Tuttolomondo et al. 2012). There is insufficient evidence that p-selectin is affected by PA (Palmefors et al. 2014; Chen et al. 2014), but, in this cohort, p-selectin was significantly lower in physically active women and, at follow-up, there was a statistically significant decrease.

CD40L, a marker of platelet activation, is a protein originating in T-cells and endothelial cells that promotes mononuclear cell recruitment. CD40L is thought to mediate functions crucial to the development of atherosclerosis (Tuttolomondo et al. 2012). The present study showed a reduction in CD40L during follow-up, suggesting associations between this agent and PA. Scientific evidence for this association is lacking and is only shown in populations with limited generalizability (Chen et al. 2014).

Our p-selectin and CD40L results show that PA may be advantageous in the prevention of endothelial dysfunction and, ultimately, CVD.

### Strengths and limitations of the study

Our design allowed us to examine the association between exercise levels and biomarkers and to assess individual changes during follow-up. All tests were standardized and biomarker analyses were blinded. We used a comprehensive set of biomarkers relevant for globally challenging diseases, and the relationships of several of these biomarkers to PA in healthy individuals are largely unexplored. Relatively few were lost to follow-up; in addition, the use of mixed models provides robust statistics that can account for dropouts that are missing at random, handle data dependency in repeated measures, and provide full utilization of all available observations. This strategy is advantageous compared to traditional approaches that only utilize complete data. Removing individuals with incomplete observations potentially provides more biased estimates, due to lack of robustness to missing data, and reduces power in analysis (Fitzmaurice et al. 2011).

The fact that the PA initiative was carried out by an employer should not be underestimated because it represents a “real world” intervention. Thus, the set-up, participant recruitment and the magnitude of results are similar to what could be expected when employers implement health-improving PA initiatives at work. Stronger associations could possibly be found between exercise levels and biomarkers in controlled laboratory environments, but this is not realistic in an unassisted occupational setting. Self-reported exercise levels could also weaken associations, due to misclassification, recall bias or problems with averaging the exercise levels (Ainsworth et al. 2015).

The type of setting made it difficult to include a control group, which could, under proper conditions, enable us to separate the effects of PA from other external factors. However, randomizing our cohort into control and intervention groups would not be ideal because the whole cohort showed interest in increasing their PA. Selecting a control group from those in the company initially not wanting to participate in the PA program or from another company would lead to a question on actual comparability between the groups. Instead, we chose to let the participants be their own controls, and assessed the individual changes in biomarkers.

We cannot ignore self-selection in this study. The 300 that participated in the PA program may have had other characteristics (motivation, age, health and education) than those who chose not to participate. While 74% of the company employees were road workers, only 26% of our cohort had this profession. In terms of improving the health of the company’s workers, this self-selection is not optimal, because manual laborers are known to have increased mortality, but not if they exercise during their leisure time (Clays et al. 2013). Four out of the five that were classified with metabolic syndrome did not attend the examination at follow-up. This suggests a further selection towards a more healthy population participating during the follow-up.

## Conclusions

Our work puts forward a potential benefit of PA on levels of inflammatory biomarkers, and points out that change in PA behavior leads to changes in inflammatory status. Our findings suggesting favorable relationships between PA and MCP-1, CD40L, and p-selectin should be of particular interest due to prior insufficient or lacking scientific evidence. The WHO considers CVD, diabetes and cancers amongst the most globally health-threatening diseases, and successful preventive strategies could have great economic and environmental impacts. Future work needs to establish these possible relationships and increase the understanding of underlying mechanisms.

## Electronic supplementary material

Below is the link to the electronic supplementary material.

Supplementary material 1 (DOCX 13 KB)

Supplementary material 2 (DOCX 16 KB)

Supplementary material 3 (DOCX 18 KB)

Supplementary material 4 (DOCX 14 KB)

## Abbreviations

${\dot V}{{\rm{O}}_{{\rm{2max}}}}$: Maximal oxygen uptake
PA: Physical activity
BMI: Body mass index
CRP: C-reactive protein
MCP-1: Monocyte chemoattractant protein-1
TNF-α: Tumor necrosis factor-alpha
Il-6: Interleukin-6
CD40L: CD40 ligand
