# Supplementary material for: Physical activity initiated by employer induces improvements in a novel set of biomarkers of inflammation: an 8-week follow-up study
Source: Eur J Appl Physiol. 2017 Feb 9;117(3):521–32. doi: 10.1007/s00421-016-3533-5 (PMC5346428; doi:10.1007/s00421-016-3533-5)
Supplement: Supplementary file 1 — Supplementary material 1 (DOCX 13 KB) [file 421_2016_3533_MOESM1_ESM.docx]

**Supplementary appendix A – Blood collection and serum analyses**

**Blood Collection and serum preparation**

Blood was sampled at the same time of day before and after the PA period. Blood was collected in S-Monovette 7.5 ml Serum geltubes (Sarstedt AG & Co., Nümbrecht, Germany, Cat. No.: 1602). The samples were left for at least 30 min and maximum 60 minutes at room temperature prior to 1000 xg centrifugation for 15 minutes, and being aliquoted into 500 μl volumes in 1.8 mL Nunc tubes and stored at -80°C.

**Serum analyses**

Serum levels of MCP-1, IL-6, TNF-α, Leptin, P-Selectin and CD40L were determined using seven kits of a magnetic bead-based Luminex screening 6 plex assay (Cat. no.: LXSAHM-07, Lot no.: 1386929, Biotechne, UK) and seven kits of a one plex Adiponectin assay (Cat. no.: LXSAHM-01, Lot no.: 1386925, Biotechne, UK). The serum samples were thawed on ice, vortexed and spun at 10 000xg for 10 min at 4°C and the supernatant was subsequently diluted a twofold for the 6 plex and 1:200 for the Adiponectin 1 plex assay. The sample preparation and the assays were carried out according to the manufacturer's instructions. The beads assays were processed, recorded and analyzed with a Luminex IS 100 (Bio Rad, Hercules, CA, USA) powered with the Bio-Plex manager Software version 6.0 build 617. The standard curve was performed in duplicate with recombinant proteins in a 3-fold dilution, validated by the StatLIA software package (ver. 3.2; Brendan Scientific, Inc.) with a five-parametric logistic curve-modelling and used to determine the protein levels in the serum samples. To increase the range of the standard curve an additional standard point was added. All samples were analyzed in duplicate and the average intra assay %CV for all samples was determined for MCP-1 (5.7), the IL-6 (4.0), P-selectin (2.0), CD40L (3.4), Leptin (3.4), TNF-a (3.7) and Adiponectin (2.7). An in-house longitudinal control was used on each plate (n=7) to determine the inter assay %CV for the 6 plex; MCP-1 (10.0), IL-6 (6.8), P-selectin (8.3), CD40 Ligand (11.8), Leptin (14.5) and TNF-a (7.5).
