# Supplementary material for: Physical activity initiated by employer induces improvements in a novel set of biomarkers of inflammation: an 8-week follow-up study
Source: Eur J Appl Physiol. 2017 Feb 9;117(3):521–32. doi: 10.1007/s00421-016-3533-5 (PMC5346428; doi:10.1007/s00421-016-3533-5)
Supplement: Supplementary file 2 — Supplementary material 2 (DOCX 16 KB) [file 421_2016_3533_MOESM2_ESM.docx]

**Supplementary appendix B. R and Stata commands for doing linear mixed model analyses.**

# **R implementation of linear mixed model analyses of each biomarker**

Here are the R commands for the mixed model analyses of each single biomarker that have produced the results of Table 2.

**## Description of data variables:**

**## ID: Unique person identification**

**## Exercise: Exercise category at baseline, three levels: <= 1**

**## time/wk, 2-3 times/wk, >= 4 times/wk**

**## Time: Categorical variable with two values: 1 (baseline) and**

**## 2(follow-up)**

**## Education: Categorical variable with two values: “High school”**

**## and “College/University”**

**## AgeMinus20Div10: Continuous variable, equal (age-20)/10**

**## var.gender: categorical variable input to the analysis,**

**## with values “All”, “Females”, “Males”**

**## Select gender subset if stratified by male or female, otherwise**

**## use all observations**

**if (var.gender=="Females") # female**

**ind.sub <- dat$Gender == "F"**

**else if (var.gender=="Males") # male**

**ind.sub <- dat$Gender == "M"**

**else**

**ind.sub <- rep(TRUE, nrow(dat)) # all**

**## Crossed random intercept for participant (ID) and plate**

**## (const is set to 1 for all observations)**

**random.formula <- list(const=pdBlocked(list(pdIdent(~Plate),**

**pdIdent(~ID-1))))**

**## Linear mixed model analysis of MCP1 using the lme function**

**## (identical analyses for the other biomarkers: replace MCP1**

**## by TNF-alpha,IL-6, ln(Leptin), Adiponectin, P-selectin**

**## or CD40L)**

**reg <- lme(MCP1~Exercise + Time + AgeMinus20Div10 + Education,**

**random=random.formula,data=dat[ind.sub,])**

# **Implementation of the analysis of the joint biomarker model**

We also investigated the total effect of exercise (association and change) on all biomarkers of inflammation by implementing a joint biomarker model. Prior to analysis, each biomarker were normalized to a mean = 0 and SD = 1. Since an increase in levels is favorable in adiponectin, this biomarker was reversed in the joint model by multiplying its values with -1. A reduction for the normalized joint biomarker value would then be favorable. A linear mixed model was applied with this normalized joint biomarker as outcome variable.

The analysis of the normalized joint biomarker included the steps detailed below: reshaping the data to appropriate long format, remove outliers, normalize biomarkers, and the finally analyzing the data using the lmer function in R.

**a) STATA script for reshaping the data to appropriate long format**

**## STATA script for transforming the data to the appropriate**

**## long format: one record for each biomarker (MCP-1,**

**## TNF-alpha,IL-6, ln(Leptin), Adiponectin, P-selectin**

**## CD40L**

**## Read data, one record (line) includes observation from all**

**## 7 biomarkers**

**use “/path…/biomarker_follow_up.dta", clear**

**## Add a common prefix to biomarker names**

**rename MCP1 cytokinValueMCP1**

**rename IL6 cytokinValueIL6**

**rename Pselectin cytokinValuePselectin**

**rename CD40L cytokinValueCD40L**

**rename Leptin cytokinValueLeptin**

**rename TNFa cytokinValueTNFa**

**rename Adiponectin cytokinValueAdiponectin**

**## Add an ID (1,2,3, …) for each record**

**gen tmpId=_n**

**## Reshape data such that one record are split in 7**

**## One record now includes observation from only one**

**## biomarker**

**reshape long cytokinValue@, i(tmpId) j(cytokinVar) string**

**## encode cytokinVar from string to numeric variable**

**rename cytokinVar cytokinVar_tmp**

**encode cytokinVar_tmp, generate(cytokinVar)**

**## Save data**

**saveold "/path../biomarker_multi.dta", replace version(12)**

**b) Mixed model analysis in R using the lmer function**

**## Initial steps:**

**## - Read data (the biomarker_multi.dta file) generated by the**

**## Stata script, and store them in “datmulti” (data frame object)**

**## - Remove outliers already identified for IL-6**

**## - Normalise biomarkers (MCP-1, TNF-alpha, ..) such that**

**## they each have mean=0 and SD=1.**

**## Fixed + random effects formula where**

**## - Exercise: the effect of baseline exercise on biomarker**

**## from BL to FU**

**## - Time: average change in biomarker value from BL to FU**

**## - The analysis is adjusted for age, education, smoking (no/yes), gender.**

**formula.reg <- formula("cytokinValueNorm~cytokinVar +**

**Exercise3CatBL + Time + Education + Smoking +**

**Gender:cytokinVar + Age:cytokinVar + (1|Plate) +**

**(Time|ID) + (1|ID:cytokinVar)”)**

**## Call to the lmer function for doing linear mixed model analysis**

**reg <- lmer(formula.reg, data=datmulti)**
