# Supplementary material for: Physical activity initiated by employer induces improvements in a novel set of biomarkers of inflammation: an 8-week follow-up study
Source: Eur J Appl Physiol. 2017 Feb 9;117(3):521–32. doi: 10.1007/s00421-016-3533-5 (PMC5346428; doi:10.1007/s00421-016-3533-5)
Supplement: Supplementary file 3 — Supplementary material 3 (DOCX 18 KB) [file 421_2016_3533_MOESM3_ESM.docx]

**Supplementary appendix C. The impact of BMI adjustment on separate analyses for each biomarker variable.**

|  | | | Not adjusted for BMI | | | | Adjusted for BMI | | | |
| --- | --- | --- | --- | --- | --- | --- | --- | --- | --- | --- |
| Outcome | Covariate | Category | B | 95 % CI |  | P | B | 95 % CI |  | P |
| MCP1 | Exercise | 2-3 times/week | -17.15 | -60.94 | 26.64 | 0.44 | -17.47 | -61.85 | 26.91 | 0.44 |
|  |  | >= 4 times/week | **-81.98** | **-142.92** | **-21.04** | **0.0086** | **-82.12** | **-144.17** | **-20.07** | **0.0097** |
|  | Time |  | -13.44 | -29.38 | 2.50 | 0.098 | -13.22 | -29.63 | 3.20 | 0.11 |
|  | Age |  | 20.45 | 3.37 | 37.52 | 0.019 | 19.79 | 2.34 | 37.24 | 0.026 |
|  | Gender | Female | -41.06 | -86.26 | 4.14 | 0.075 | -39.18 | -85.48 | 7.13 | 0.097 |
|  | Education | College/Uni | 15.04 | -29.09 | 59.17 | 0.5 | 15.56 | -29.95 | 61.07 | 0.5 |
|  | BMI |  |  |  |  |  | 0.34 | -5.28 | 5.96 | 0.91 |
|  | Smoking |  | -9.47 | -77.51 | 58.58 | 0.78 | -9.35 | -77.62 | 58.91 | 0.79 |
| TNFa | Exercise | 2-3 times/week | -0.72 | -2.29 | 0.86 | 0.37 | -0.61 | -2.20 | 0.98 | 0.45 |
|  |  | >= 4 times/week | 0.36 | -1.83 | 2.56 | 0.74 | 0.47 | -1.76 | 2.70 | 0.68 |
|  | Time |  | -0.26 | -0.85 | 0.32 | 0.37 | -0.27 | -0.86 | 0.33 | 0.38 |
|  | Age |  | 0.36 | -0.25 | 0.98 | 0.25 | 0.31 | -0.32 | 0.93 | 0.34 |
|  | Gender | Female | -2.77 | -4.40 | -1.15 | 0.00091 | -2.64 | -4.30 | -0.99 | 0.0019 |
|  | Education | College/Uni | -1.53 | -3.11 | 0.06 | 0.059 | -1.38 | -3.01 | 0.25 | 0.096 |
|  | BMI |  |  |  |  |  | 0.07 | -0.14 | 0.27 | 0.52 |
|  | Smoking |  | 2.34 | -0.10 | 4.79 | 0.06 | 2.34 | -0.10 | 4.79 | 0.06 |
| IL6 | Exercise | 2-3 times/week | -0.04 | -0.49 | 0.41 | 0.85 | 0.04 | -0.40 | 0.47 | 0.87 |
|  |  | >= 4 times/week | -0.06 | -0.69 | 0.57 | 0.85 | 0.11 | -0.51 | 0.72 | 0.74 |
|  | Time |  | -0.13 | -0.33 | 0.08 | 0.24 | -0.13 | -0.35 | 0.08 | 0.22 |
|  | Age |  | 0.22 | 0.05 | 0.40 | 0.014 | 0.17 | -0.01 | 0.34 | 0.058 |
|  | Gender | Female | -0.31 | -0.77 | 0.16 | 0.19 | -0.17 | -0.62 | 0.29 | 0.47 |
|  | Education | College/Uni | -0.50 | -0.95 | -0.05 | 0.03 | -0.36 | -0.81 | 0.08 | 0.11 |
|  | BMI |  |  |  |  |  | 0.08 | 0.03 | 0.14 | 0.0041 |
|  | Smoking |  | 0.83 | 0.13 | 1.53 | 0.02 | 0.83 | 0.16 | 1.50 | 0.016 |
| logLeptin | Exercise | 2-3 times/week | **-0.39** | **-0.68** | **-0.09** | **0.011** | **-0.25** | **-0.48** | **-0.01** | **0.038** |
|  |  | >= 4 times/week | **-0.69** | **-1.10** | **-0.28** | **0.0012** | **-0.44** | **-0.77** | **-0.12** | **0.0076** |
|  | Time |  | 0.01 | -0.06 | 0.08 | 0.82 | 0.02 | -0.04 | 0.09 | 0.49 |
|  | Age |  | 0.13 | 0.01 | 0.25 | 0.027 | 0.06 | -0.03 | 0.15 | 0.19 |
|  | Gender | Female | 1.01 | 0.70 | 1.31 | 8.3e-10 | 1.23 | 0.99 | 1.47 | 3.3e-19 |
|  | Education | College/Uni | -0.33 | -0.63 | -0.03 | 0.03 | -0.09 | -0.33 | 0.15 | 0.45 |
|  | BMI |  |  |  |  |  | 0.13 | 0.10 | 0.16 | 3e-16 |
|  | Smoking |  | -0.14 | -0.60 | 0.33 | 0.57 | -0.15 | -0.51 | 0.21 | 0.41 |
| Adiponectin | Exercise | 2-3 times/week | -0.20 | -1.51 | 1.11 | 0.77 | -0.40 | -1.70 | 0.90 | 0.55 |
|  |  | >= 4 times/week | 0.57 | -1.25 | 2.39 | 0.54 | 0.22 | -1.60 | 2.03 | 0.81 |
|  | Time |  | 0.12 | -0.13 | 0.36 | 0.36 | 0.10 | -0.16 | 0.35 | 0.46 |
|  | Age |  | 0.32 | -0.19 | 0.83 | 0.22 | 0.41 | -0.10 | 0.92 | 0.12 |
|  | Gender | Female | 3.17 | 1.82 | 4.52 | 6.6e-06 | 2.89 | 1.53 | 4.24 | 4e-05 |
|  | Education | College/Uni | 0.41 | -0.90 | 1.73 | 0.54 | 0.08 | -1.25 | 1.41 | 0.9 |
|  | BMI |  |  |  |  |  | -0.18 | -0.34 | -0.03 | 0.023 |
|  | Smoking |  | 0.38 | -1.66 | 2.42 | 0.71 | 0.39 | -1.62 | 2.40 | 0.7 |
| Pselectin | Exercise | 2-3 times/week | 1.42 | -3.96 | 6.80 | 0.6 | 2.09 | -3.24 | 7.43 | 0.44 |
|  |  | >= 4 times/week | -4.38 | -11.89 | 3.12 | 0.25 | -3.15 | -10.64 | 4.34 | 0.41 |
|  | Time |  | **-2.33** | **-3.78** | **-0.87** | **0.0019** | **-2.24** | **-3.76** | **-0.72** | **0.004** |
|  | Age |  | 2.88 | 0.76 | 4.99 | 0.0078 | 2.49 | 0.38 | 4.61 | 0.021 |
|  | Gender | Female | -9.70 | -15.24 | -4.16 | 0.00068 | -8.64 | -14.20 | -3.09 | 0.0025 |
|  | Education | College/Uni | -1.14 | -6.55 | 4.27 | 0.68 | -0.01 | -5.45 | 5.44 | 1 |
|  | BMI |  |  |  |  |  | 0.64 | -0.03 | 1.30 | 0.063 |
|  | Smoking |  | 0.51 | -7.87 | 8.89 | 0.91 | 0.47 | -7.77 | 8.70 | 0.91 |
| CD40L | Exercise | 2-3 times/week | -163.47 | -937.25 | 610.30 | 0.68 | -69.81 | -828.52 | 688.91 | 0.86 |
|  |  | >= 4 times/week | -903.74 | -2000.46 | 192.98 | 0.11 | -772.28 | -1852.51 | 307.95 | 0.16 |
|  | Time |  | **-718.14** | **-1368.26** | **-68.01** | **0.031** | **-656.93** | **-1329.08** | **15.21** | **0.055** |
|  | Age |  | 129.72 | -173.26 | 432.70 | 0.4 | 136.44 | -164.92 | 437.80 | 0.37 |
|  | Gender | Female | 72.61 | -723.11 | 868.32 | 0.86 | 52.09 | -736.53 | 840.72 | 0.9 |
|  | Education | College/Uni | -196.96 | -977.13 | 583.21 | 0.62 | -9.13 | -789.42 | 771.15 | 0.98 |
|  | BMI |  |  |  |  |  | 64.93 | -31.70 | 161.57 | 0.19 |
|  | Smoking |  | -315.98 | -1509.37 | 877.41 | 0.6 | -390.87 | -1546.77 | 765.03 | 0.51 |
