# Supplementary material for: Physical activity initiated by employer induces improvements in a novel set of biomarkers of inflammation: an 8-week follow-up study
Source: Eur J Appl Physiol. 2017 Feb 9;117(3):521–32. doi: 10.1007/s00421-016-3533-5 (PMC5346428; doi:10.1007/s00421-016-3533-5)
Supplement: Supplementary file 4 — Supplementary material 4 (DOCX 14 KB) [file 421_2016_3533_MOESM4_ESM.docx]

**Supplementary appendix D. The impact of BMI on joint model of biomarkers.**

|  | | | Not adjusted for BMI | | | | Adjusted for BMI | | | |
| --- | --- | --- | --- | --- | --- | --- | --- | --- | --- | --- |
| Covariate | Category | Cytokine | B | 95 % CI |  | P | B | 95 % CI |  | P |
| Exercise | 2-3 times/week |  | -0.089 | -0.267 | 0.089 | 0.33 | -0.040 | -0.204 | 0.124 | 0.63 |
|  | >= 4 times/week |  | -0.341 | -0.590 | -0.092 | **0.0085** | -0.259 | -0.489 | -0.028 | **0.03** |
| Time |  |  | -0.101 | -0.171 | -0.032 | **0.0052** | -0.097 | -0.170 | -0.024 | **0.01** |
| BMI |  |  |  |  |  |  | 0.047 | 0.026 | 0.068 | 2.6e-05 |
| Education | College/Uni |  | -0.166 | -0.345 | 0.013 | 0.072 | -0.088 | -0.255 | 0.079 | 0.3 |
| Smoking |  |  | 0.078 | -0.199 | 0.355 | 0.58 | 0.076 | -0.176 | 0.329 | 0.55 |
| Age |  | MCP1 | 0.014 | 0.000 | 0.027 | 0.043 | 0.010 | -0.003 | 0.023 | 0.12 |
|  |  | TNFa | 0.011 | -0.002 | 0.024 | 0.11 | 0.008 | -0.005 | 0.020 | 0.24 |
|  |  | IL6 | 0.016 | 0.003 | 0.029 | 0.015 | 0.013 | 0.000 | 0.026 | 0.049 |
|  |  | Leptin | 0.010 | -0.003 | 0.023 | 0.12 | 0.007 | -0.006 | 0.020 | 0.28 |
|  |  | Adiponectin | -0.010 | -0.023 | 0.004 | 0.15 | -0.013 | -0.025 | 0.000 | 0.054 |
|  |  | Pselectin | 0.018 | 0.005 | 0.031 | 0.0064 | 0.015 | 0.002 | 0.028 | 0.02 |
|  |  | CD40L | 0.004 | -0.009 | 0.017 | 0.54 | 0.001 | -0.011 | 0.014 | 0.83 |
| Gender | Female | MCP1 | -0.272 | -0.603 | 0.060 | 0.11 | -0.190 | -0.515 | 0.135 | 0.25 |
|  | Female | TNFa | -0.761 | -1.092 | -0.429 | 8.3e-06 | -0.682 | -1.007 | -0.357 | 4.4e-05 |
|  | Female | IL6 | -0.330 | -0.661 | 0.002 | 0.052 | -0.259 | -0.584 | 0.066 | 0.12 |
|  | Female | Leptin | 1.014 | 0.682 | 1.345 | 3.5e-09 | 1.089 | 0.764 | 1.414 | 1e-10 |
|  | Female | Adiponectin | -0.860 | -1.191 | -0.528 | 5e-07 | -0.791 | -1.116 | -0.466 | 2.2e-06 |
|  | Female | Pselectin | -0.512 | -0.844 | -0.180 | 0.0026 | -0.440 | -0.765 | -0.115 | 0.0082 |
|  | Female | CD40L | 0.109 | -0.223 | 0.441 | 0.52 | 0.179 | -0.146 | 0.504 | 0.28 |

Adjusted for gender (All), age, education and smoking (Males and All). The effect of age and gender was allowed to vary with cytokine variable. A separate intercept value was included for each cytokine variable (not shown).
